# Experimental Imaging Study of Encephalomalacia Fluid-Attenuated Inversion Recovery (FLAIR) Hyperintense Lesions in Posttraumatic Epilepsy

**DOI:** 10.1155/2021/2678379

**Published:** 2021-10-31

**Authors:** Dan Wang, Kai Shang, Zheng Sun, Yue-Hua Li

**Affiliations:** Institute of Diagnostic and Interventional Radiology, The Sixth Affiliated People's Hospital, Shanghai Jiao Tong University, No. 600, Yi Shan Road, Shanghai 200233, China

## Abstract

This study introduced new MRI techniques such as neurite orientation dispersion and density imaging (NODDI); NODDI applies a three-compartment tissue model to multishell DWI data that allows the examination of both the intra- and extracellular properties of white matter tissue. This, in turn, enables us to distinguish the two key aspects of axonal pathology—the packing density of axons in the white matter and the spatial organization of axons (orientation dispersion (OD)). NODDI is used to detect possible abnormalities of posttraumatic encephalomalacia fluid-attenuated inversion recovery (FLAIR) hyperintense lesions in neurite density and dispersion. *Methods*. 26 epilepsy patients associated with FLAIR hyperintensity around the trauma encephalomalacia region were in the epilepsy group. 18 posttraumatic patients with a FLAIR hyperintense encephalomalacia region were in the nonepilepsy group. Neurite density and dispersion affection in FLAIR hyperintense lesions around encephalomalacia were measured by NODDI using intracellular volume fraction (ICVF), and we compare these findings with conventional diffusion MRI parameters, namely, fractional anisotropy (FA) and apparent diffusion coefficient (ADC). Differences were compared between the epilepsy and nonepilepsy groups, as well as in the FLAIR hyperintense part and in the FLAIR hypointense part to try to find neurite density and dispersion differences in these parts. *Results*. ICVF of FLAIR hyperintense lesions in the epilepsy group was significantly higher than that in the nonepilepsy group (*P* < 0.001). ICVF reveals more information of FLAIR(+) and FLAIR(-) parts of encephalomalacia than OD and FA and ADC. *Conclusion*. The FLAIR hyperintense part around encephalomalacia in the epilepsy group showed higher ICVF, indicating that this part may have more neurite density and dispersion and may be contributing to epilepsy. NODDI indicated high neurite density with the intensity of myelin in the FLAIR hyperintense lesion. Therefore, NODDI likely shows that neurite density may be a more sensitive marker of pathology than FA.

## 1. Introduction

Posttraumatic epilepsy is a recurrent and chronic brain dysfunction syndrome secondary to traumatic injury of the brain. Posttraumatic epilepsy has recurrent seizures after one week of the TBI; it is one of the most serious complications of brain trauma and is characterized by repeated epileptic seizures caused by abnormal discharge of neurons [1, 2].

Extensive nerve cell necrosis, interstitial edema, and inflammatory reactions occur in brain trauma; subsequently, the neuronal cells disappear, forming encephalomalacia. As there are no nerve cells in encephalomalacia, the lesion itself does not cause epilepsy. However, the scars (composed of glial cell hyperplasia, crossing fiber bundles, and different-thickness fiber bundles) surrounding the encephalomalacia may affect the normal electrophysiological activity of neurons and cause hyperplastic glial dysfunction, which in turn may contribute to abnormal discharge leading to seizures [1, 2].

MRI can be used to study the volume, signal intensity, and adjacent structural changes of encephalomalacia. Structural MRI studies have shown encephalomalacia after brain contusion, but glial cell hyperplasia and crossing fiber bundles could not be detected. To further understand these macro- and microstructural changes, it is necessary to investigate the underlying alterations in the tissue properties of white matter. Diffusion tensor imaging (DTI) provides sensitivity to tissue microstructure but lacks specificity for individual tissue microstructure features [3, 4]. Moreover, DTI provides simple markers such as mean diffusivity (MD) and FA; however, these markers are inherently nonspecific [3, 4].

A new diffusion MRI technique of NODDI was used in this research. NODDI could estimate the microstructural complexity of dendrites and axons *in vivo* on clinical MRI scanners. Such indices of neurites relate more directly to and provide more specific markers of brain tissue microstructure than standard indices from DTI such as FA. NODDI enables mapping by combining a three-compartment tissue model with a two-shell high-angular resolution diffusion imaging (HARDI) protocol optimized for clinical feasibility [2, 5–7]. An index of OD is defined to characterize the angular variation of neurites. Studies had confirmed that NODDI-based estimation of diffusion ellipsoids in rat optical myelin staining intensity and electron microscopy dendritic architecture findings was more accurate than DTI [4]. Moreover, it has been proven that neurite morphology is a key marker of brain changes such as development and aging [4, 8, 9]. NODDI could show the OD index of neurites and hence could quantify the bending and fanning of axons, which is useful for mapping brain connectivity [4, 8, 9]. NODDI is a clinically feasible technique for *in vivo* neurite OD and density imaging. Quantifying neurite morphology in terms of its density and OD provides a window into the structural basis of brain function both in normal populations and in populations with brain disorders [10]. Changes in neurite morphology are additionally implicated in numerous neurological disorders such as multiple sclerosis, amyotrophic lateral sclerosis, and Alzheimer's disease [11–13]. Some studies have provided that DTI is a more specific measure of axonal/myelin loss; DTI-derived neurite density may better reflect the overall white matter burden in epilepsy [14]. There are some articles discussing the relationship between epilepsy and the density of nerve protrusions, such as focal cortical dysplasia in patients with epilepsy and temporal lobe epilepsy patients with hippocampal sclerosis [1, 15]. But there are not that many articles discussing the relationship between the density of neurite density and the FLAIR hyperintense lesions surrounding the brain softening lesions. This is the purpose of this article.

In this experimental study, we used NODDI, a clinically feasible technique, for *in vivo* density imaging using intracellular volume fraction (ICVF) and neurite OD to explore the imaging features of encephalomalacia in patients with posttraumatic epilepsy. This may have certain clinical functions in the preventive treatment of epilepsy and the reduction of the incidence of epilepsy after trauma.

## 2. Material and Methods

### 2.1. Patients Enrolled

**Figure 1:**
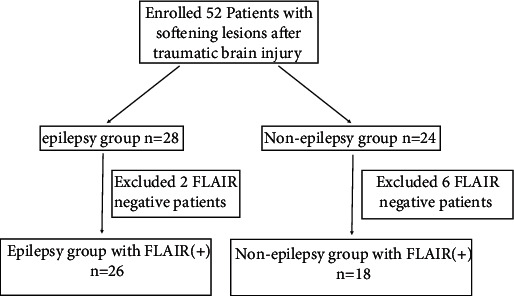
Flowchart. (1) Epilepsy group: after excluding two patients with FLAIR-based hypointensity, 26 patients with epilepsy with FLAIR hyperintensity were enrolled. (2) Nonepilepsy group: after excluding six patients with FLAIR-based hypointensity, we recruited 18 patients with no epilepsy.

After excluding two patients with FLAIR-based hypointensity, we enrolled 26 patients with epilepsy who experienced brain trauma more than three months ago and showed FLAIR images of hyperintensity around encephalomalacia (epilepsy group). Epilepsy is caused by brain injury; it was diagnosed based on International League Against Epilepsy (ILAE) (2017). As controls, after excluding six patients with FLAIR-based hypointensity, we recruited 18 patients with no epilepsy, and three months after brain trauma, they were also associated with FLAIR hyperintensity around encephalomalacia (nonepilepsy group) (Figure 1, Table 1). All participants enrolled underwent MRI scans.

The inclusion criteria were as follows: (1) posttraumatic epilepsy (seizures after 7 days of trauma) according to ILAE Classification 2017 and mild-to-moderate brain injury (Glasgow Coma Scale (GCS) score 9–15); (2) typical clinical symptoms of seizures; (3) follow-up time after trauma of more than three months; (4) encephalomalacia seen on MRI; and (5) electroencephalography (EEG) with or without typical spike, spike and slow waves, and paroxysmal slow waves. Participants in the control group had no clinical symptoms of seizures.

The exclusion criteria were as follows: patients with a history of epilepsy before trauma or with a family history of epilepsy or with a history of febrile seizures or with a significant medical history of acute encephalitis, meningitis, ischemic encephalopathy, or suspicious epileptogenic lesions (e.g., tumors, cortical dysplasia, central nervous system infections, or vascular malformation) or those with metal implantation in their body.

### 2.2. MRI

A 3T MRI scanner (MAGNETOM, Verio, Siemens Healthcare, Erlangen, Germany) with a 32-channel head coil was used. The imaging sequences included conventional MR sequences (T1-weighted imaging, T2-weighted imaging, and FLAIR) and DWI sequences. For T1 3D axial images, we have the following: field of view (FOV), 230 mm; repetition time/echo time (TR/TE), 1500/2.96 ms; flip angle, 98; voxel size, 0.9 × 0.9 × 0.1 mm^3^; slice thickness, 1 mm; and distance factor, 50%. DWI was performed for five *b* values (*b* = 0, 500, 1000, 1500, and 2000 s/mm^2^) and 30 directions. We have the following: FOV, 230 mm; TR/TE, 5100/109 ms; voxel size, 1.8 × 1.8 × 3 mm^3^; matrix size, 128 × 128; slice thickness, 3 mm; distance factor, 30%; and number of slices, 35.

### 2.3. MR Image Measurement

#### 2.3.1. Softening Volume

Brain encephalomalacia volume was measured on T1 3D sequence images, along with reference to the T2W FLAIR sequence image. Manual measurement of softening volume was mainly based on axial images using the two-dimensional measurement method, i.e., single-layer area and layer thickness multiplied to obtain single-layer volume and layer-by-layer volume. The total volume of the lesion was added using the following formula: *V* = (*S*_1_ + *S*_2_+⋯*S*_*n*_) × (layer thickness + slice gap), where *V* represents the absolute volume of encephalomalacia and *S* represents the area of the encephalomalacia measured by each layer (Table 2).

#### 2.3.2. NODDI Processing (ICVF, OD, FA, and ADC)

**Figure 2:**
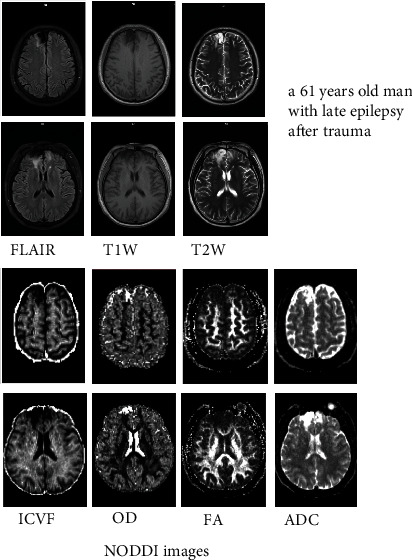
A 61-year-old man had late epilepsy that occurred six months after trauma. He had an encephalomalacia with FLAIR hyperintense lesions in both frontal lobes (FLAIR, T1W and T2W images, and NODDI ICVF, OD, FA, and ADC images).

**Figure 3:**
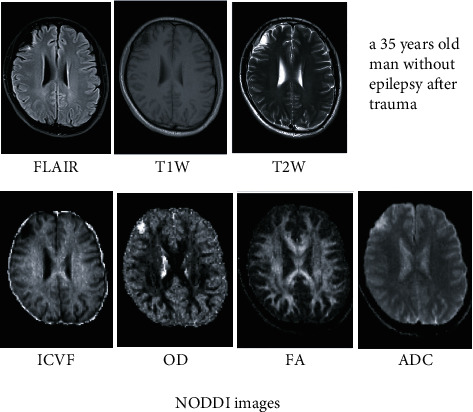
A 35-year-old man without epilepsy one year after trauma. He had softening foci with FLAIR hyperintense lesions in the right frontal lobe (FLAIR, T1W and T2W images, and NODDI ICVF, OD, FA, and ADC images).

**Figure 4:**
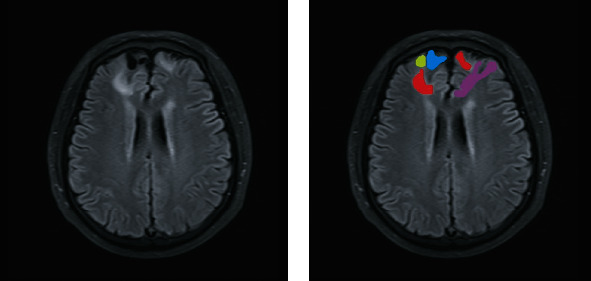
A FLAIR image ROI label example of a 61-year-old man with late epilepsy six months after trauma. Four colors were used: red-labeled FLAIR hyperintense lesion surrounding encephalomalacia; green-labeled FLAIR hypointense lesion surrounding encephalomalacia; blue-labeled encephalomalacia; and purple-labeled contralateral area and adjacent normal part.

The DWI DICOM images with different *b*-shell configurations were converted to NIfTI format using dcm2niix, and the preprocessing pipeline of DWI includes (1) denoise, (2) eddy correction, and (3) coregistration of all *b* values of DWI into *b* = 0 s/mm^2^. The denoise algorithm explores the random field theory and uses the Principal Component Analysis (PCA) to exploit the data redundancy to finally denoise the DWI. The eddy current correction and motion correction were all performed within FSL, where eddy current was based on outlier detection and the slice-to-volume movement model and where a rigid motion correction was applied. All the preprocessing was executed in MRtrix3 with FSL as backend [7, 16, 17]. After the preprocessing, the DWI were then fitted in a NODDI model and the ICVF, OD, FA, and ADC maps for each participant were generated using the AMICO Python package [18] (Figures 2 and 3). A radiologist with 10 years of experience drew four types of regions of interest (ROIs) for each participant in the FLAIR axial images (including intracellular volume fraction (ICVF), OD, FA, and apparent diffusion coefficient (ADC)) slice by slice, and ROI positions are adjusted accordingly in the coronal and sagittal images. One ROI covered the FLAIR(+) part, one ROI covered the FLAIR(-) part, one ROI covered the encephalomalacia, and one ROI defined as the normal part covered the contralateral area or adjacent normal part of the same patient (Figure 4). The ROIs drawn on FLAIR images were then transferred to NODDI using the binarized linear interpretation-based resampling method, and then the NODDI metrics were measured directly from NODDI-derived maps.

### 2.4. Statistical Analysis

These diffusion parameters (ICVF, OD, FA, and ADC values) are the mean values within the drawn ROIs:

1. A two-sample *t*-test was performed to compare ICVF values in the FLAIR hyperintense part of the epilepsy and nonepilepsy groups and to compare the OD, FA, and ADC values
2. A two-sample *t*-test was also performed to compare the FLAIR hypointense part of the epilepsy and nonepilepsy groups and to compare the OD, FA, and ADC values
3. The ICVF/OD/FA/ADC values were compared between the three parts (FLAIR(+) part, FLAIR(-) part, and normal part) of the epilepsy group by using one-way analysis of variance (ANOVA) and Fisher's exact test. The Bonferroni test was used to correct the intergroup comparisons made using the two-sample *t*-test (i.e., FLAIR(+) part vs. FLAIR(-) part, FLAIR(+) part vs. normal part, and FLAIR(-) part vs. normal part). ANOVA and the Bonferroni exact test were also performed between the three parts of the nonepilepsy group

## 3. Results

The encephalomalacia volume and FLAIR(+) lesion volume of the epilepsy group were significantly larger than those of the nonepilepsy group (Table 2).

### 3.1. ICVF

**Figure 5:**
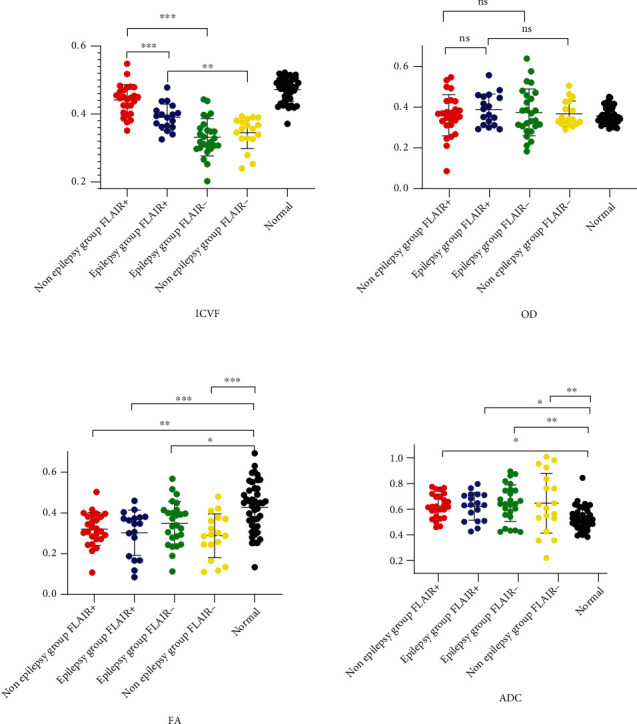
(a) ICVF. (b) OD. (c) FA. (d) ADC.

**Figure 6:**
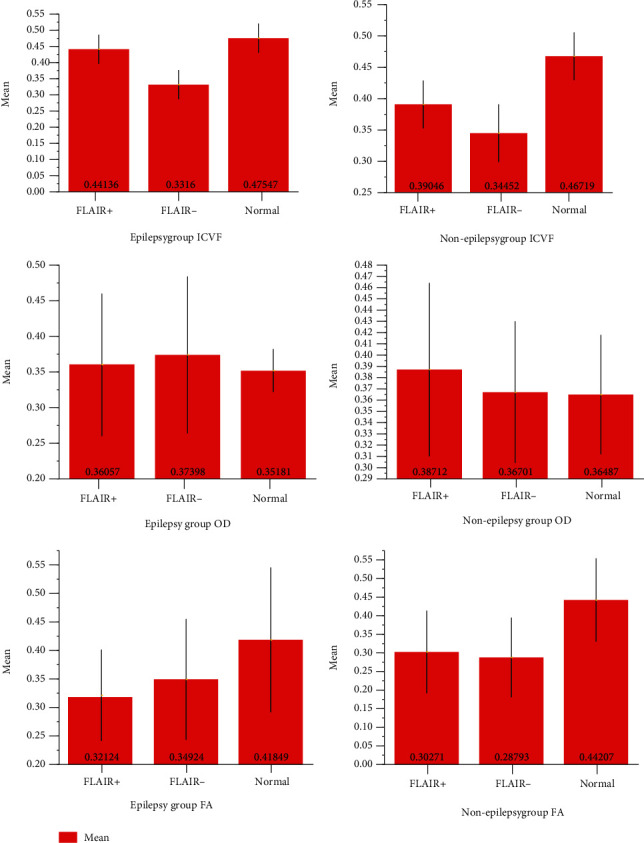
(a) ICVF: FLAIR hyperintense part: the FLAIR hyperintense part of the epilepsy group is significantly higher; epilepsy group: the FLAIR(+) part is significantly higher than the FLAIR(-) part but is significantly lower than the normal part. (b) OD. (c) FA: the normal part is significantly higher than the FLAIR(+) part and FLAIR(-) part in both the epilepsy and nonepilepsy groups, but there is no significant difference between the FLAIR(+) part and the FLAIR(-) part. (d) ADC: the FLAIR hyperintense part and FLAIR hypointense part are significantly higher than the normal part in both the epilepsy and nonepilepsy groups.

*(1) FLAIR Hyperintense Part*. The FLAIR hyperintense part of the epilepsy group is significantly higher than that of the nonepilepsy group (*t* = 3.919, *P* < 0.001) (Figures 5 and 6, Table 3).

*(2) FLAIR Hypointense Part*. There was no significant difference in the FLAIR hypointense part of the epilepsy and nonepilepsy groups (*t* = −0.817, *P* = 0.419) (Table 3).

*(3) Epilepsy Group*. There was a significant difference between the three parts of the tissue around encephalomalacia: the FLAIR(+) part was significantly higher than the FLAIR(-) part (*P* < 0.001), but the FLAIR(+) part was significantly lower than the normal part (*P* = 0.024) (Table 4).

*(4) Nonepilepsy Group*. There was a significant difference between the three parts of the tissue around encephalomalacia: the FLAIR(+) part was significantly higher than the FLAIR(-) part (*P* = 0.005), but the FLAIR(+) part was significantly lower than the normal part (*P* = 0.001) (Table 4).

### 3.2. OD

*(1) FLAIR Hyperintense Part*. There was no significant difference in the FLAIR hyperintense part of the epilepsy and nonepilepsy groups (*t* = −0.940, *P* = 0.353) (Figures 5 and 6, Table 3).

*(2) FLAIR Hypointense Part*. There was no significant difference in the FLAIR hypointense part of the epilepsy and nonepilepsy groups (*t* = 0.235, *P* = 0.816) (Table 3).

*(3) Epilepsy Group*. There was no significant difference in the epilepsy group or nonepilepsy group between the three parts of the tissue around encephalomalacia (Table 4).

*(4) Nonepilepsy Group*. There was no significant difference in the OD value of the FLAIR(+) part and FLAIR(-) part and normal part of either of the groups (Table 4).

### 3.3. FA

*(1) FLAIR Hyperintense Part*. There was no significant difference in the FLAIR hyperintense part of the epilepsy group and nonepilepsy group (*t* = 0.643, *P* = 0.524) (Figures 5 and 6, Table 3).

*(2) FLAIR Hypointense Part*. There was no significant difference in the FLAIR hypointense part of the epilepsy group and nonepilepsy group (*t* = 1.871, *P* = 0.069) (Table 3).

*(3) Epilepsy Group*. The FA comparison between the FLAIR(+) part and the normal part has a significant difference (*P* = 0.001).

*(4) Nonepilepsy Group*. The normal part was significantly higher than the FLAIR(+) and FLAIR(-) parts.

### 3.4. ADC

*(1) FLAIR Hyperintense Part*. There was no significant difference in the FLAIR hyperintense part of the epilepsy group and nonepilepsy group (*t* = 0.093, *P* = 0.927) (Figures 5 and 6, Table 3).

*(2) FLAIR Hypointense Part*. There was no significant difference in the FLAIR hypointense part of the epilepsy group and nonepilepsy group (*t* = −0.003, *P* = 0.998) (Table 3).

*(3) Epilepsy Group*. The normal part was significantly lower than the FLAIR(+)and FLAIR(-) parts (Table 3).

*(4) Nonepilepsy Group*. The FLAIR hyperintense and hypointense parts were significantly higher than the normal part (Table 3).

## 4. Discussion

In this study, in the epilepsy group, NODDI indicated high neurite density with the intensity of myelin in the FLAIR hyperintense lesion, demonstrating a correlation of high neurite density and epilepsy.

### 4.1. Relationship between NODDI and the Mechanism of Epilepsy

Many studies have shown that local mossy fiber sprouted around brain contusion lesions. Mossy fiber germination is associated with increased epileptic activity after brain trauma, suggesting that mossy fiber germination may be related to the occurrence of epilepsy [19, 20]. And these are some studies about NODDI findings in epilepsy. Winston et al. studied refractory epilepsy patients with focal cortical dysplasia, and they concluded that NODDI could pervade tissue microstructure information, including ICVF as a marker of neurite density; NODDI may therefore assist the clinical identification and localization of focal cortical dysplasia in patients with epilepsy [21]. Sone et al. studied temporal lobe epilepsy patients with and without hippocampal sclerosis using NODDI, and they found decreased neurite density mainly in the temporal areas of temporal lobe epilepsy patients [1]. Winston et al. studied patients with refractory temporal lobe epilepsy, and they concluded that diffusivity changes in grey and white matter are primarily related to reduced neurite density in the temporal pole [15]. Neurite density may represent a more sensitive and specific biomarker of progressive neuronal damage in refractory temporal lobe epilepsy [15].

This study experimentally attempted to explore changes in the microstructure of encephalomalacia in patients with posttraumatic epilepsy. The NODDI technique was used in this study to describe changes in the number of neurite orientation dispersion and density in patients with posttraumatic late epilepsy.

In this study, the characteristics of late epilepsy caused by posttraumatic encephalomalacia showed some characteristic properties. Morphological abnormalities alone could not fully describe encephalomalacia enough. Many epileptogenic lesions do not show morphological abnormalities on conventional MRI. Just as in the formation of posttraumatic encephalomalacia in this study, gliosis can be found in the conventional MRI sequence, but NODDI showed more details in the FLAIR parts of encephalomalacia in epilepsy and nonepilepsy groups.

In the epilepsy group, the ICVF value of the FLAIR hyperintense part was significantly higher than that of the nonepilepsy group, indicating that the FLAIR hyperintense part may have more dendrites. However, OD did not show significant differences between the three parts (FLAIR(+) part, FLAIR(-) part, and normal part) or the two groups. Although more dendrites are present in the FLAIR hyperintense part, the spatial organization of the axons (OD) may be very complicated. FA values of FLAIR hyperintense and hypointense parts were significantly lower than those of the normal parts in both groups. In contrary to FA, ADC values of FLAIR hyperintense and hypointense parts were significantly higher than those of normal parts in both groups. However, neither FA nor ADC FLAIR hyperintense parts showed differences between the epilepsy and nonepilepsy groups. The FLAIR high-signal hyperintense parts of the epilepsy and nonepilepsy groups were different from the FLAIR hypointense part components. In this study, the encephalomalacia of the epilepsy group was significantly larger than that of the nonepilepsy group, and the FLAIR hyperintense volume of the epilepsy group was also larger than the FLAIR hyperintense volume of the nonepilepsy group. We speculated that the larger the volume of the encephalomalacia, the more severe the loss of brain tissue and the more likely it is to cause epilepsy.

### 4.2. NODDI and Neurite Density

NODDI is based on non-Gaussian water molecule movement theory and describes water in the intracellular space bound, for example, by axonal or dendritic membranes. The differentiation of intra- and extracellular water forms the basis of measuring neurite morphology via diffusion MRI [7, 22].

NODDI offers the opportunity to extend the application of neurite morphology quantification from being confined within the realm of postmortem histology to becoming a part of routine clinical practice. Jespersen et al.'s study showed a strong correlation of neurite density with the intensity of myelin staining under light microscopy, indicating that neurite density may be a useful marker for demyelination disorders [4]. The weaker dependence of FA on neurite density further suggests that neurite density may be a more sensitive marker of pathology than FA and may highlight early signs of demyelination before FA [3, 6, 23].

In this study, we used the NODDI technique to evaluate the FLAIR hyperintense tissue surrounding the encephalomalacia, based on the theory of proliferation of glial cells, neurite density, and entanglement of synapses after brain trauma [24, 25]. Studies have shown that the mechanisms of posttraumatic seizures are complex, with reactive astrocyte proliferation, increased neurite density, microglial proliferation, and release of neurotrophic factors; all of these could affect the sprouting of nerve fiber branches and increase neurite density. The neurite density and the direction increased, which may be related to the formation and repetition of excitatory circuits during epilepsy and which may participate in the formation of local scar tissue.

Our study limitations are as follows. Not all FLAIR hyperintense areas around the encephalomalacia are responsible for epilepsy. Thus, further research with a larger sample size is needed to validate the results. The age difference between the epilepsy and nonepilepsy groups may affect the NODDI results.

## 5. Conclusion

This study is an experimental study that used the MRI NODDI technique to study epilepsy and softening FLAIR hyperintense lesions. NODDI indicated high neurite density with the intensity of myelin in the FLAIR hyperintense lesion, demonstrating a correlation of high neurite density and epilepsy. NODDI may further suggest that neurite density may be a more sensitive marker of pathology than FA. NODDI can offer an opportunity to extend the application of neurite morphology quantification in routine clinical practice.

## Figures and Tables

**Table 1:** Clinical demographics of patients with TLE.

		Epilepsy group	Nonepilepsy group
Age at examination (years)		40.538 ± 11.315	48.556 + 7.254
Sex (no.), men : women		17 : 9	10 : 8
Lesion	Frontal lobe	17	13
	Temporal lobe	9	5
Focus side	Left	11	7
	Right	10	8
	Bilateral	5	3
Disease duration after trauma (months)		8.923 ± 3.52	9.556 ± 3.185
Duration of seizure (months)		5.423 ± 3.313	4.944 ± 3.038
GCS		11.038 ± 1.28	11.556 ± 1.199

**Table 2:** Volume comparison of 26 FLAIR-positive patients from the epilepsy group and 18 FLAIR-positive patients from the nonepilepsy group.

	Epilepsy group with FLAIR(+)		Nonepilepsy group with FLAIR(+)		Comparison between groups
	Mean	SD	Mean	SD	*P* value
Number	26		18		
Encephalomalacia volume (cm^−3^)	29.172	12.88	19.890	6.545	0.008
Volume of FLAIR(+) lesions	8.607	3.821	5.814	2.414	0.009

**Table 3:** NODDI parameter comparison of FLAIR hyperintense lesions in the epilepsy group and nonepilepsy group.

FLAIR(+) part		Lesion				Comparison between the epilepsy and nonepilepsy groups
		Epilepsy group		Nonepilepsy group		*P* value
		Mean	SD	Mean	SD	
Number		26		18		
ICVF	FLAIR(+)	0.441	0.045	0.390	0.038	<0.001^∗^
	FLAIR(-)	0.332	0.055	0.345	0.046	0.419
	Normal part	0.475	0.033	0.467	0.038	0.446
OD	FLAIR(+)	0.363	0.101	0.387	0.077	0.353
	FLAIR(-)	0.374	0.114	0.367	0.063	0.816
	Normal part	0.352	0.031	0.36487	0.053	0.306
FA	FLAIR(+)	0.321	0.080	0.303	0.111	0.524
	FLAIR(-)	0.349	0.106	0.288	0.107	0.069
	Normal part	0.418	0.127	0.442	0.112	0.529
ADC	FLAIR(+)	0.624	0.093	0.621	0.108	0.927
	FLAIR(-)	0.646	0.143	0.647	0.233	0.998
	Normal part	0.543	0.096	0.508	0.074	0.145

∗ indicates that the difference of the means is significant at the 0.05 level.

**Table 4:** NODDI parameter comparison of FLAIR hyperintense lesions and FLAIR hypointense lesions in both the epilepsy and nonepilepsy groups.

Number		Lesion						Comparison between the FLAIR(+) part, the FLAIR(-) part, and the normal part		
Epilepsy group		FLAIR(+) part		FLAIR(-) part		Normal part		P1	P2	P3
		Mean	SD	Mean	SD	Mean	SD	*P* value	*P* value	*P* value
ICVF	26	0.441	0.045	0.332	0.055	0.475	0.033	<0.001^∗^	0.024^∗^	<0.001^∗^
OD		0.361	0.101	0.374	0.114	0.352	0.031	1	1	1
FA		0.321	0.080	0.349	0.106	0.418	0.127	1	0.001^∗^	0.065
ADC		0.624	0.093	0.646	0.143	0.543	0.096	1	0.034^∗^	0.004^∗^
Nonepilepsy group		FLAIR(+) part		FLAIR(-) part		Normal part		P1	P2	P3
		Mean	SD	Mean	SD	Mean	SD	*P* value	*P* value	*P* value
ICVF	18	0.390	0.038	0.345	0.046	0.467	0.038	0.005^∗^	<0.001^∗^	<0.001^∗^
OD		0.387	0.077	0.367	0.063	0.365	0.053	1	0.926	1
FA		0.303	0.111	0.288	0.107	0.442	0.112	1	0.001^∗^	<0.001^∗^
ADC		0.621	0.108	0.647	0.233	0.508	0.074	1	0.096	0.009^∗^

P1: comparison between the FLAIR(+) part and the FLAIR(-) part; P2: comparison between the FLAIR(+) part and the normal part of the same patient; P3: comparison between the FLAIR(-) part and the normal part of the same patient. ∗ indicates that the difference of the means is significant at the 0.05 level.

## Data Availability

The data used to support the findings of this study are available from the corresponding author upon request.
